# Interactions Between Plastic, Microbial Biofilms and *Gammarus pulex*: An Initial Investigation

**DOI:** 10.1007/s00128-021-03448-5

**Published:** 2022-01-06

**Authors:** Katey L. Valentine, Alistair B. A. Boxall

**Affiliations:** Department of Environment and Geography, University of York, Heslington, York, YO10 5DD UK

**Keywords:** Plastic pollution, *Gammarus*, Microbial biofilm, Chemoattraction

## Abstract

There is increasing evidence that microbial biofilms which form on the surface of marine plastics can increase plastics palatability, making it more attractive to organisms. The same information, however, does not exist for freshwater systems. This study observed the response of the freshwater amphipod *Gammarus pulex* when exposed to 3 cm-diameter discs of biofilm-covered plastic, both alone and when presented alongside its natural food. *G. pulex* did not fragment or consume the plastic materials, and the presence of colonised plastic in the immediate environment did not alter the amount of time organisms spent interacting with their natural food. This study provides baseline information for virgin and microbially colonised low-density polyethylene and polylactic acid film. Further studies, with other types of plastic possessing different physical properties and with different microbial biofilm compositions are now required to build further understanding of interactions between plastic, microbial biofilms, and freshwater shredding invertebrates.

**Supplementary Information:**

The online version contains supplementary material available at 10.1007/s00128-021-03448-5.

It is now well-known that plastic pollution is ubiquitous throughout natural aquatic systems globally, and that a wide variety of organisms commonly interact with and ingest the plastic material they encounter in their environment (Davidson and Dudas 2016; Horton et al. 2018). Freshwater environments, such as rivers and streams are often the first recipients of plastic emissions and they act as a source to the world’s oceans (Vaid et al. 2021). Despite the abundance of plastic waste in freshwater systems (Lu et al. 2021), they have traditionally received far less research attention than marine environments (Horton et al. 2017; Meng et al. 2020), leaving many key questions about the interactions of freshwater organisms with plastic waste unanswered.

As with other types of hard surfaces, when plastic enters natural water, microorganisms such as bacteria, algae and fungi (Zettler et al. 2013; Amaral-Zettler et al. 2020) rapidly colonise its surface. Over time, these microorganisms accumulate, forming a distinct layer over the plastic surface—referred to as biofilm. Although findings in the literature remain mixed, there is evidence that under certain conditions the microbial composition of the biofilm on plastic surfaces can be significantly distinct from that of other natural surfaces such as wood and glass (e.g. Kirstein et al. 2018; Oberbeckmann et al. 2018). Once formed, biofilm communities can significantly change the physical properties of plastic from its virgin state, such as by altering particle buoyancy in the water column (Kaiser et al. 2017) or by modifying material properties such as crystallinity and stiffness (McGivney et al. 2020). There is also mounting evidence that these biofilms can actively influence the way in which aquatic organisms interact with plastic in their environment. For example, it is thought that biofilms can alter the plastic’s chemosensory signature by emitting compounds such as dimethyl sulfide—which is naturally produced by certain types of algae and bacteria and is known to be a strong feeding stimulant in the marine environment (Savoca et al. 2016). Whilst one study found that the presence of a biofilm reduced the ingestion rate of microplastics by a species of hard coral (Allen et al. 2017), many other studies have found that biofilms can increase attraction and palatability of plastic to marine organisms from many types of functional feeding groups (Savoca et al. 2017; Vroom et al. 2017; Hodgson et al. 2018; Porter et al. 2019; Pfaller et al. 2020; Weideman et al. 2020). This attraction to plastic-associated biofilms may have a range of consequences including: increasing exposure of the organisms to plastic additives (Rochman et al. 2013); the organism expending their energy budget on ingestion of material with lower calorific value than their natural food; and providing a mechanism for microplastic creation (Hodgson et al. 2018). Additionally, laboratory plastic-exposure studies which do not account for these biofilms may be misrepresenting the relationship that organisms have with plastic materials in their natural habitat.

Although this attraction to plastic-associated biofilms has been shown for a variety of marine species, similar studies do not exist for freshwater systems; the ability of the biofilm to alter the interactions between plastic and freshwater invertebrates therefore remains undocumented. In this study, we present an investigation into the interactions of the freshwater amphipod *Gammarus pulex* with flexible plastic films. A similar study was conducted by Hodgson et al. (2018) who found that the common marine amphipod *Orchestia gammarellus* readily shredded three different types (high-density polyethylene, undefined degradable, undefined biodegradable) of 1 cm^2^ virgin and biofilm-colonised plastic film, with significantly more shredding seen in colonised plastic treatments. *G. pulex* is widespread in rivers across Europe and is a common model organism for ecotoxicology studies (Weber et al. 2018). These amphipods are shredding detritivores; they use their toothed mandibles to shred and consume food such as leaves and other plant material (Mateos-Cárdenas et al. 2020) and, like other *Gammarus* sp., show a strong dietary preference for material which has a microbial biofilm on its surface (Bärlocher and Kendrick 1975; Bloor 2011). It is thought that *G. pulex* may feed on plant material only as a means to access the nutritional microorganisms on its surface (Nelson 2011), and they may therefore have interest in other non-plant materials with a microbial biofilm on their surface. In natural environments a significantly higher abundance of *Gammarus* sp. have been found associated with anthropogenic litter compared to natural rock (Wilson et al. 2021) and in behaviour experiments *G. pulex* has demonstrated clear attraction to the chemosensory signature of microbial biofilms (Lange et al. 2005). Previous studies have demonstrated the ingestion of biofilm-free microplastics 10–150 μm in size by *G. pulex* and other gammarids (Weber et al. 2018; Mateos-Cárdenas et al. 2020), but their interaction with larger, microbially colonised plastic remains unexplored.

The primary aim of this study was to determine whether, with no other food sources available, the freshwater amphipod *G. pulex* will shred macro-sized pieces of virgin or biofilm-colonised plastic film, in a similar manner to that observed in the marine shredder *O.gammarellus.* It was hypothesized that *G. pulex* will shred colonised LDPE and PLA films to feed on the attached microbial community and some shredding may also be seen to a lesser extent on un-colonised virgin material. The secondary aim was to determine whether the colonised plastic is attractive to *G. pulex* when other food sources are available and whether its presence interferes with the normal feeding behaviour of *G. pulex* on natural food. It was hypothesized that the colonised plastic will be somewhat attractive to *G. pulex*, that they will show behavioural interest in it, and over time they will consume less of their natural food than when there is colonised plastic present.

## Materials and Methods

Low-density polyethylene (LDPE) and poly lactic acid (PLA) films, derived from commercially available bags were used in this experiment. LDPE film was 45 μm thick and cut from plastic sold as carrier bags. PLA film was 40 μm thick and cut from plastic sold to hold food items. The FTIR spectra of these materials was obtained using a Nicolet iS10 spectrometer (Fig. S1) and used to confirm their polymer identity. To prepare plastic for microbial colonisation, sheets of plastic were cut and attached inside a 240 × 240 × 130 mm custom-built stainless-steel woven mesh (0.57 mm aperture) cage. Healthy *Acer pseudoplatanus* leaves were obtained from trees in a semi-rural location (53° 47′ 01.6″ N 1° 21′ 59.4″ W) and air-dried for 3 weeks before being attached inside cages with the plastic. Cages were placed in the surface water of the River Ouse upstream of York city centre, UK (54° 00′ 30.7″ N 1° 11′ 28.7″ W). After 3 weeks, the cages were removed from the river, plastic and leaves were rinsed with milli-Q water and cut into 3 cm diameter discs. During these 3 weeks, virgin discs of LDPE and PLA of the same size were soaked in sterile milli-Q water in the dark at 15°C in order to control for any changes that may have occurred to the plastic due to water absorption. Five discs from each of the treatments were used to quantify the weight of biofilm attached to the plastic and three discs from each treatment were imaged under a microscope to visualize the plastic surface and biofilm—details of these methods are given in Supplementary Table S2.

*G. pulex* were collected from a small stream in Bishop Wilton, UK (53° 59′ 07.9″ N 0° 47′ 08.6″ W) using a kick sampling method and their identity was confirmed with an taxonomic key (Dobson 2012). They were transported back to the laboratory and maintained at 15°C under a 12:12 diurnal cycle in an aerated 50 L glass acclimation tank containing river water for at least 1 week prior to experimental testing. During this time, they were fed ad libitum with commercially available Tetra® crustacean food. The river water was collected from the same site in the River Ouse where cages were placed; this water was used unfiltered for the acclimation, but before its use in experiments was freshly collected and filtered to 0.7 μm with glass fibre filters.

Two separate feeding experiments and one behaviour experiment were conducted. For all experiments, amphipods were a mixed population of males and non-egg-bearing females ≥ 10 mm in length and were starved for 48 h prior to the start of all experiments. For the two feeding experiments each organism was placed in an individual glass jar containing 150 mL of filtered river water and maintained under the same temperature and light conditions as during acclimation. Jars were aerated with a glass pipette for 15 min each day to maintain a high dissolved oxygen concentration in the water. For the first feeding experiment, to determine if *G. pulex* would shred plastic, one plastic disc was placed in a jar with each amphipod for 5 days. This experiment consisted of four treatment groups: LDPE colonised; LDPE virgin; PLA colonised and PLA virgin (n = 10 for each group). Ten control jars for each treatment were also set up with the same conditions but without *G. pulex*. Both virgin and colonised LDPE material was positively buoyant and therefore remained at the air–water interface. All PLA material was negatively buoyant and sat at the bottom of the jar. The second feeding experiment was designed to determine the effect of the presence of microbially colonised plastic on the amphipod’s natural food consumption over 3 days. For this experiment there were three treatment groups, with amphipods given either: a single leaf disc; a leaf disc and colonised LDPE disc; a leaf disc and colonised PLA disc (n = 9) for each group. Ten control jars containing one leaf disc but no amphipod were also run in parallel. At the end of both feeding experiments plastic and leaf discs were removed, rinsed, and stored at − 80°C until they were imaged to quantify their surface area. Plastic discs were scanned using an Epson ET-2720 scanner and leaf discs were imaged using an Olympus TG-5 camera. The surface area of plastic and leaf discs was then calculated using ImageJ version 1.53a using the thresholding tool. Plastic discs were examined under a stereo microscope to look for bite marks or other visual evidence of plastic shredding.

For the behaviour experiment, a single amphipod was transferred to a 10.5 cm diameter low form cylindrical glass beaker filled with 600 mL of aerated river water and placed in a 15°C environmental cabinet in dim light and allowed to acclimate for 1 h. After this, one plastic and one leaf square (1 × 1 cm), held with a metal clip, were placed at either end of the glass beaker ~ 3 cm apart and the behaviour of the amphipod was video recorded for 15 min with an iPhone 6 camera set ~ 30 cm above the beaker. Treatment groups for this experiment consisted of: leaf and colonised LDPE; leaf and colonised PLA; leaf and an empty clip holding no material, (n = 9 for each treatment). The amount of time that amphipods spent on each material type and the number of visits they made to each material was determined. The time that amphipods spent not in contact with any material was also recorded and is referred to as ‘swimming time’.

Statistical analysis and plot construction were carried out in R studio Version 1.2.1335. All data was examined for normality and homogeneity of variances with either parametric or non-parametric tests carried out based on the outcome. Details of specific statistical tests and data transformations carried out are outlined in Supplementary Table S3. The significance level was set at 0.05.

## Results and Discussion

**Fig. 1:**
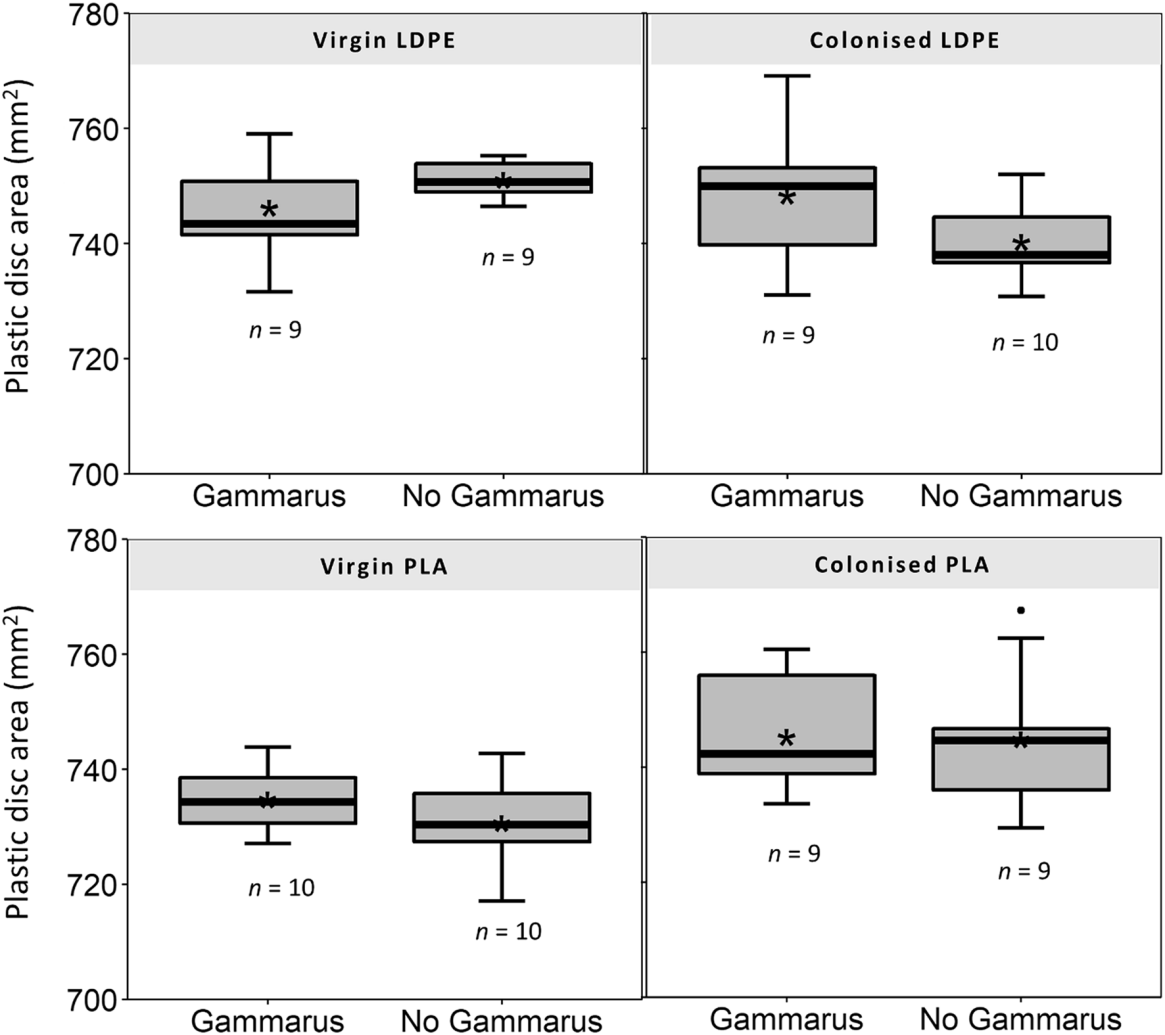
Surface area of virgin and microbially-colonised LDPE and PLA discs after 5 days under treatment conditions. Box plots show control treatments where no *G. pulex *was present compared to experimental treatments where plastic was exposed to *G. pulex. *Asterisks (*) symbols represent the mean average for each treatment with the number of replicates shown below each box

**Fig. 2:**
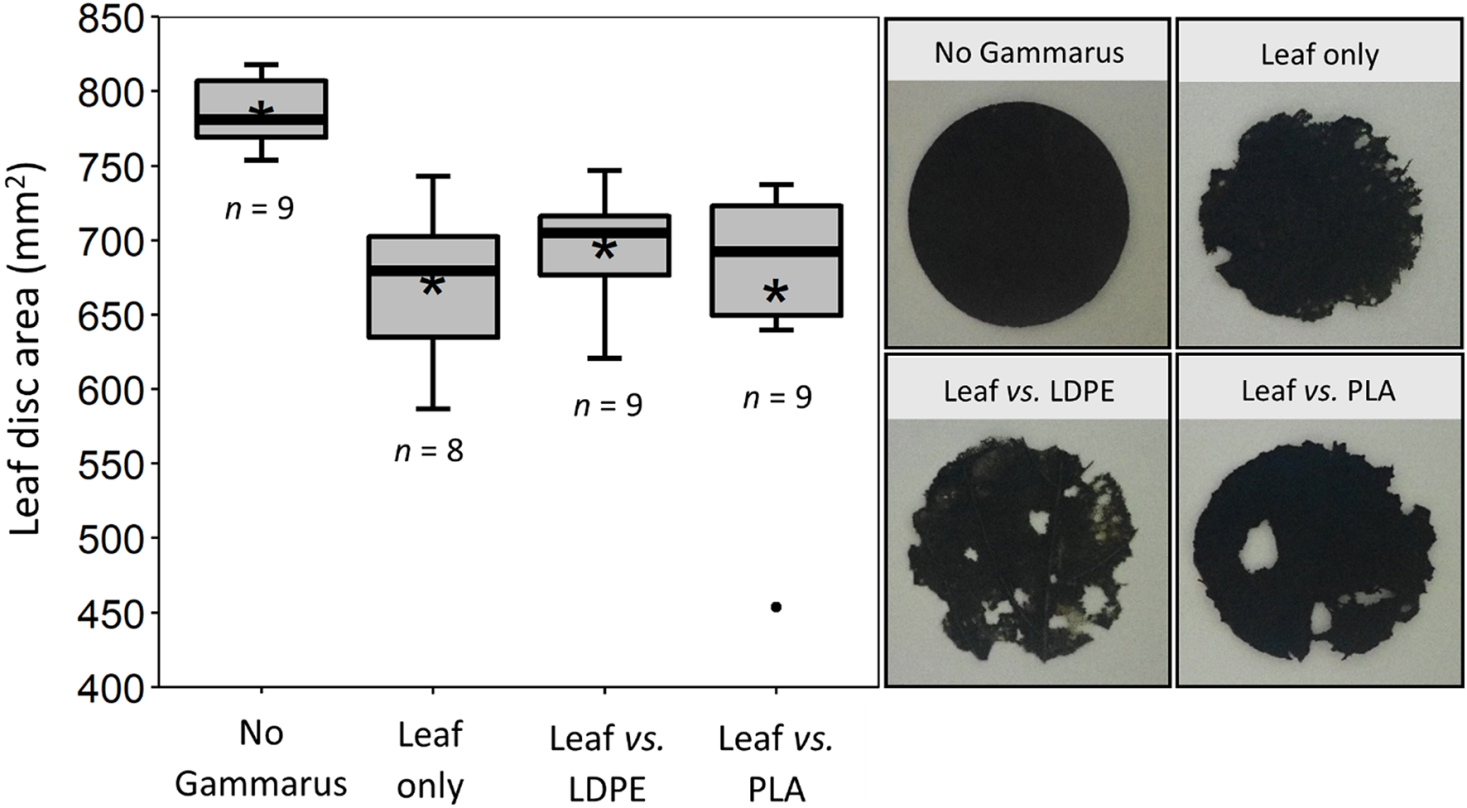
Plot on left shows the surface area of leaf disc after 3 days in each treatment. The ‘No Gammarus’ treatment was the control treatment to compare against other experimental treatments. Asterisks (*) show the average for each treatment, outliers are represented by black circles and the number of replicates for each treatment is shown below each box. Images on the right show examples of a typical leaf disc for each treatment after 3 days

**Fig. 3:**
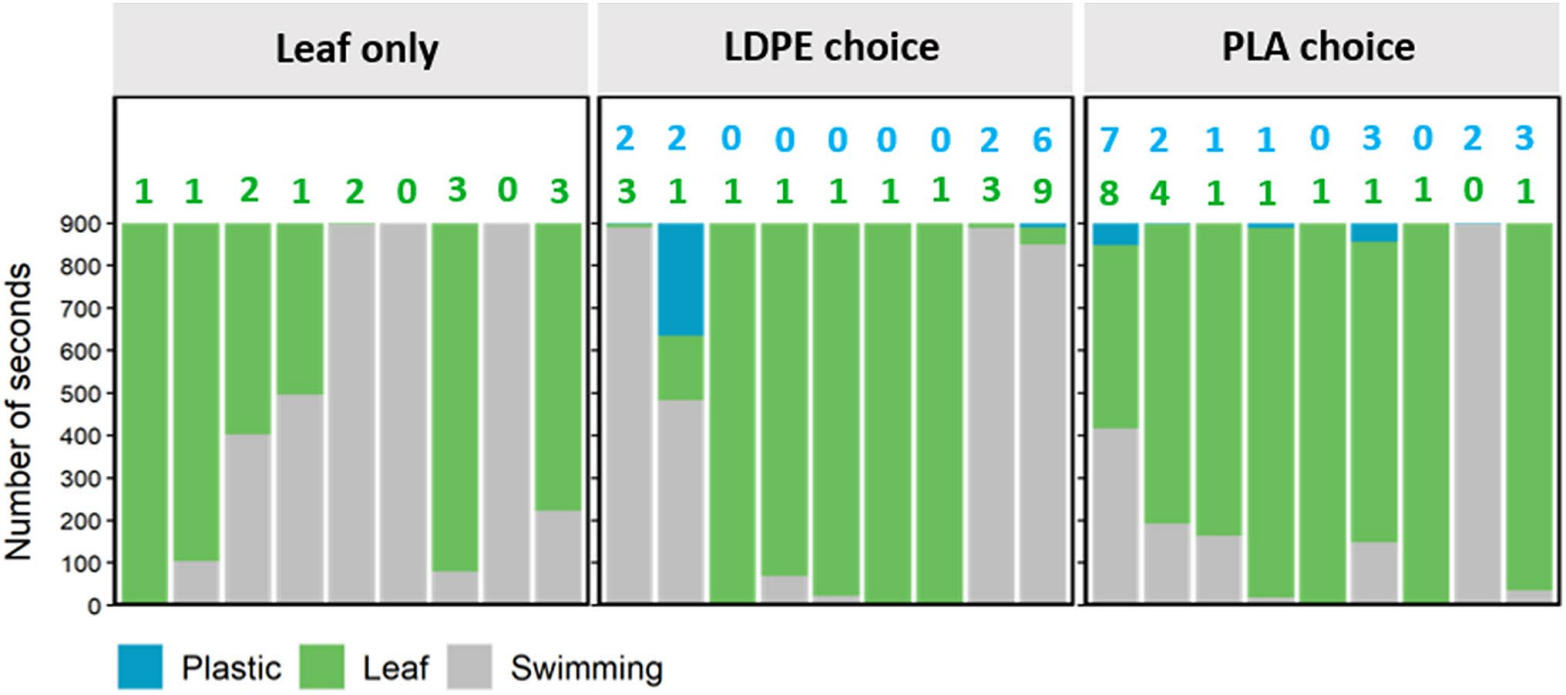
Total amount of time that *G. pulex *spent in contact with leaf, LDPE or PLA, or swimming over the 15-min (900 s) observation period. Each bar represents one replicate for the given treatment. Numbers above bars indicate the number of separate visits that the amphipod made to each material (leaf visits in green, plastic visits in blue) for the given replicate

The average weight of biofilm attached to LDPE and PLA material was found to be 120 ± 44.72 and 73.3 ± 22.36 µg cm^−2^ respectively. Imaging under the microscope clearly showed the presence of a biofilm compared to controls with organisms such as diatoms and green algae attached to the surface (Figs. S4 and S5). In the first feeding experiment no significant difference in the surface area of plastic discs was found between control discs and discs that were exposed to *G. pulex* for 5 days for any of the treatments (Fig. 1). Virgin LDPE (*p* = 0.152), colonised LDPE (*p* = 0.103), virgin PLA (*p* = 0.191), colonised PLA (*p* = 0.949); averages and standard deviations of treatments are given in Table S6. Examination of discs under the microscope also showed no evidence that amphipods had shredded or bitten the plastic. After the 3-day leaf feeding experiment there was a visually obvious consumption of leaf discs by amphipods for all treatment groups where *G. pulex* was present, and no noticeable changes for the control group with no *G. pulex* (Fig. 2). These observations were reflected in the surface area measurements, with a significant difference in disc area found between groups (*p* < 0.001). Post-hoc statistical comparisons revealed the difference to be due to the larger surface area of the control treatment which had an average calculated disc area of 786 ± 24 mm^2^ and was significantly larger than the leaf-only treatment 672 ± 53 mm^2^_,_ the leaf vs. LDPE treatment 696 ± 36 mm^2^ and the leaf vs. PLA treatment 667 ± mm^2^ with all *p* values < 0.001. There were no significant differences found between leaf-only treatments and the LDPE-choice (*p =* 0.556) or PLA choice (*p* = 0.743) treatments, or between the two choice treatments (*p* = 0.743). In the behaviour experiment there was a considerable amount of variability in the response of amphipods between replicates (Fig. 3). Between the three treatment groups no significant differences were found in the time that amphipods spent in contact with leaf material (*p =* 0.335), with an average of 455 ± 374, 513 ± 439 and 679 ± 295 s spent on leaf material for leaf-only, LDPE-choice and PLA-choice treatments respectively. The average number of leaf visits also did not differ significantly between these treatments (*p* = 0.792) or the amount of time spent swimming (*p* = 0.284). For treatments where colonised plastic was presented alongside the leaf, contact with plastic was hugely variable between replicates with an average of 30 ± 88 s and 12 ± 20 s contact time for LDPE and PLA treatments with no significant difference found between the time spent on each plastic type (*p* = 0.379) or the number of visits to each plastic type (*p* = 0.543).

The absence of any plastic shredding indicates that these types of macro-plastic films which pollute freshwater systems (Wilson et al. 2021) are unlikely to be fragmented by *G. pulex* in the same manner that they are in marine systems by the amphipod *O. gammarellus* (Hodgson et al. 2018) and the first hypothesis of this study can therefore be rejected. However, it should be considered that these conclusions may only be valid for plastic with a toughness and thickness the same or greater than that of the tested materials. Previous studies have found a significant correlation between leaf toughness and *G. pulex* feeding rate—with tougher leaves being consumed at a lower rate than softer ones (Foucreau et al. 2013). Although the actual toughness values were not measured in this study, colonised leaf material during the experiments was observed to be much softer, and pulled apart with much less mechanical force compared to colonised LDPE and PLA which remained physically robust when handled. It is therefore possible that *G. pulex* was physically unable to shred these LDPE and PLA films with its mandibles. In natural environments plastic is subjected to various weathering processes. These include photooxidation, mechanical abrasion, hydrolysis and biodegradation, and over extended periods (months–years) these processes can result in physical alteration of the plastic with it often becoming more brittle and easily fragmented (Song et al. 2017). In the future, to tease apart whether it was the physical toughness of the plastic, or the lack of chemical similarity to natural leaf material which resulted in a lack of interaction in this study, in further studies it would be interesting to explore whether *G. pulex* would fragment less tough plastic films—such as those weakened by prolonged environmental weathering.

It is well documented that *G. pulex* feeds by shredding and consuming the substrate and attached biofilm together, compared to organisms such as *Asellus aquaticus* which scrapes microbial biofilms off the surface of material to feed (Graça et al. 1993). Despite this, it is unknown whether in some circumstances *G. pulex* may feed directly on the biofilm itself without shredding the matrix it is attached to—and it is therefore possible that individuals could have accessed the biofilm directly in this way during this study. It may also be the case that *G. pulex* simply did not perceive the colonised plastic as food material and therefore did not attempt to fragment or consume it in any way. Although there was a clear biofilm present on the plastic surface after 3 weeks in the river, a larger and thicker biofilm (with a potentially different microbial composition) is likely to have developed if incubated for a longer time. It would therefore be interesting to carry out further studies to determine whether the size and composition of the biofilm may influence the response of *G. pulex.*

When *G. pulex* was presented with colonised plastic alongside natural food for 3 days, there was no evidence to indicate that the presence of colonised plastic interfered with the normal feeding behaviour of the organism. These findings are in contrast to what was observed in the sea urchin *Paracentrotus lividus*, which readily fragmented colonised plastic in the presence of its normal food (Porter et al. 2019). In comparison to the present study, *P. lividus* capably fragmented even virgin macro-plastics when no other food was present—confirming that the material was not too tough for them to deal with and that they appeared to have little selectivity when searching for food. Results from the short-term behaviour portion of the study also provided no significant evidence for the attraction of *G. pulex* to the colonised plastic and the second hypothesis for the study can therefore also be rejected. Nevertheless, individuals from four of the nine LDPE replicates and seven of the nine PLA replicates did make visits to the plastic at some point during the exposure period and in one of the LDPE-choice replicates the amphipod spent over four minutes in contact with the plastic with its feeding appendages orientated onto the plastic–biofilm surface. For one of the PLA-choice replicates the amphipod repeatedly went back and forth between the PLA and leaf before finally settling on and feeding on the leaf. It remains unclear however, whether the plastic interactions observed were indicative of food-searching behaviour or whether the amphipods were simply exploring materials until they felt safe enough to begin feeding. The clear preference for leaf material seen in this study is similar to previous studies with other Gammarid species which also exhibited strong preferences for some food types over others (e.g. Pellan et al. 2016). It is unclear whether the leaf material in this study was chemically more attractive to the amphipods or whether it was simply less tough than plastic and easier for the organism to rapidly consume. A further study which presented these choices with materials enclosed in fine mesh to prevent amphipods having contact with them [in a similar manner as Lange et al. (2005)] would help to distinguish which of these factors is driving the results seen in this study. These findings may suggest that the significant associations seen between *G. pulex* and anthropogenic materials observed in rivers (Wilson et al. 2021) may be due to the ability of these materials to provide physical shelter, rather than a food-driven attraction of *G. pulex* to the attached biofilms.

Taken together, these results show that *G. pulex* is unlikely to fragment structurally intact colonised plastic films present in its environment and that the presence of microbially colonised plastic in its immediate environment does not appear to significantly alter the way they interact with their natural food. These results represent findings for two types of plastic film in their virgin form and in one stage of microbial colonisation, and therefore further questions regarding different material types and toughness, and biofilm compositions remain. This study provides a broad initial exploration of the interactions between plastic, microbial biofilms and a freshwater invertebrate, which until now, have remained unexamined. These findings set an initial point of reference for future research to build on to address outstanding questions such as the role of biofilm thickness and composition in influencing *G. pulex* interactions, and whether there is a link between the physical toughness of plastic and the *G. pulex* shredding behaviour observed.

## Supplementary Information

Below is the link to the electronic supplementary material.

Supplementary material 1 (DOCX 8044.7 kb)
