# Supplementary material for: Interactions Between Plastic, Microbial Biofilms and Gammarus pulex: An Initial Investigation
Source: Bull Environ Contam Toxicol. 2022 Jan 6;108(4):609–15. doi: 10.1007/s00128-021-03448-5 (PMC8979863; doi:10.1007/s00128-021-03448-5)
Supplement: Supplementary file 1 — Supplementary material 1 (DOCX 8044.7 kb) [file 128_2021_3448_MOESM1_ESM.docx]

**Figure S1** ART-FTIR spectra of virgin LDPE (A) and PLA (B) film material used in this study. Spectra were collected at The Centre for Ecology and Hydrology, Wallingford, using a Nicolet iS10 spectrometer. Data was collected between 450 and 4000 cm^1^ at a resolution of 4cm^-1^ with 32 sample and background scans. Commercially sourced LDPE bag material was found to closely match the spectra obtained from consumer LDPE material given in Huppertsberg and Knepper (2020). Commercially sourced PLA bag material was found to closely match spectra the obtained from pure PLA pellets given in Yuniarto et al. (2016).


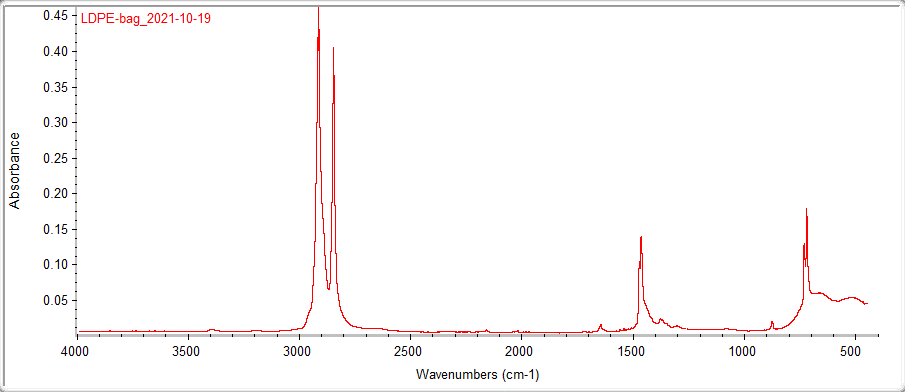

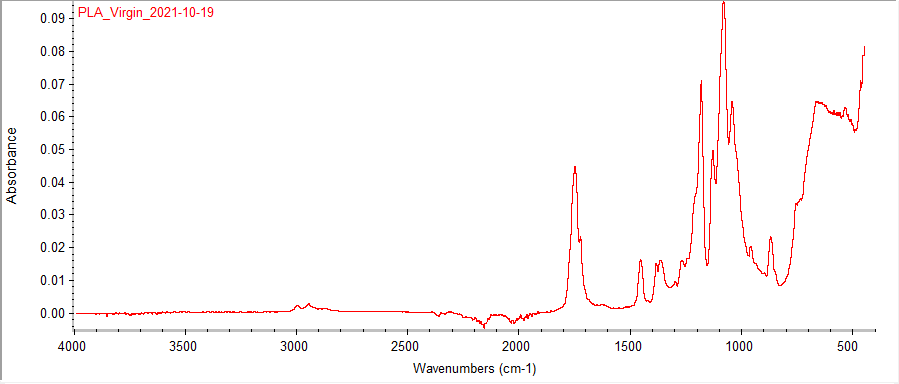


**A**

**B**

**Table S2** Average weight change of dried LDPE and PLA coupons before and after surface brushing to remove attached biofilms. To ensure that weight changes in colonised samples that had been incubated in a river for three weeks was due to the biofilm weight, the same methods were also applied to control virgin samples which had been incubated in sterile Milli-Q water for the same time period and did not have a microbial biofilm on their surface. Coupons of colonised and virgin plastic were dried in an oven at 30°C for three hours before being weighed using an analytical balance. The biofilm was then removed from samples using a soft natural bristle brush and Milli-Q water; samples were checked under the microscope to ensure complete removal of the biofilm. The surface of control samples was brushed in the same way to replicate methods. Coupons were then re-dried and re-weighed and the change in weight per unit area was calculated. One virgin LDPE and one virgin PLA sample showed a weight decrease of 0.0001 grams and one virgin PLA sample showed an increase of 0.0001 grams with all other virgin samples maintaining the same weight. Weight changes in colonised samples are therefore determined to be due to the weight of the attached biofilm.

| Sample | Average weight change (µg/cm^2^) |
| --- | --- |
| Colonised LDPE | 120 ± 44.72 |
| Virgin LDPE | 2.69 ± 6.01 |
| Colonised PLA | 73.3 ± 22.36 |
| Virgin PLA | -0.05 ± 9.64 |

**Table S3** details of all statistical tests carried out for the three experiments in the study.

| **Comparison** | **Details** | **Test** |  |
| --- | --- | --- | --- |
| *Plastic disc area* | | |  |
| Virgin LDPE: Gammarus *vs.* no Gammarus | Data passed Shapiro-wilk normality but not equal variance even after transformation | Welch’s t-test |  |
| Colonised LDPE: Gammarus *vs.* no Gammarus | Data passed Shapiro-wilk normality and equal variance. Performed Welch’s t-test to maintain consistency and comparable results | Welch’s t-test |  |
| Virgin PLA: Gammarus *vs.* no Gammarus | Data passed Shapiro-wilk normality and equal variance. Performed Welch’s t-test to maintain consistency and comparable results | Welch’s t-test |  |
| Colonised PLA: Gammarus *vs.* no Gammarus | Data passed Shapiro-wilk normality and equal variance. Performed Welch’s t-test to maintain consistency and comparable results | Welch’s t-test |  |
| *Leaf disc area* | | | |
| Area of leaf dics between different treatments after three-day exposure period | Data did not pass Shapiro-wilk normality test even after transforming data using square root and cube root functions. | Kruskal Wallis test.  Followed by a post hoc pairwise Wilcoxon test with a Benjamini-Hochberg (BH) adjustment for multiple comparisons |  |
| *Behaviour experiment* | | |  |
| Time spent on leaf between all three treatments | Normality of residuals could not be achieved so non-parametric test conducted | Kruskal Wallis test |  |
| Number of visits to leaf between all three treatments | Normality was achieved though a cube root transformation. However, normality could not be achieved for the number of plastic visits data. To maintain consistency and allow better comparisons a non-parametric test has been carried out for both comparisons. | Kruskal Wallis test |  |
| Time spent swimming between all three treatments | Transformation achieved normality but non-parametric test carried out to maintain consistency between statistical comparisons within the same experimental comparisons | Kruskal Wallis test |  |
| Time spent on plastic between LDPE choice and PLA choice treatments | Transformation achieved normality but non-parametric test carried out to maintain consistency between statistical comparisons within the same experimental comparisons | Kruskal Wallis test |  |
| Number of visits to plastic between LDPE choice and PLA choice  treatments | Normality of residuals could not be achieved | Wilcoxon signed rank test |  |


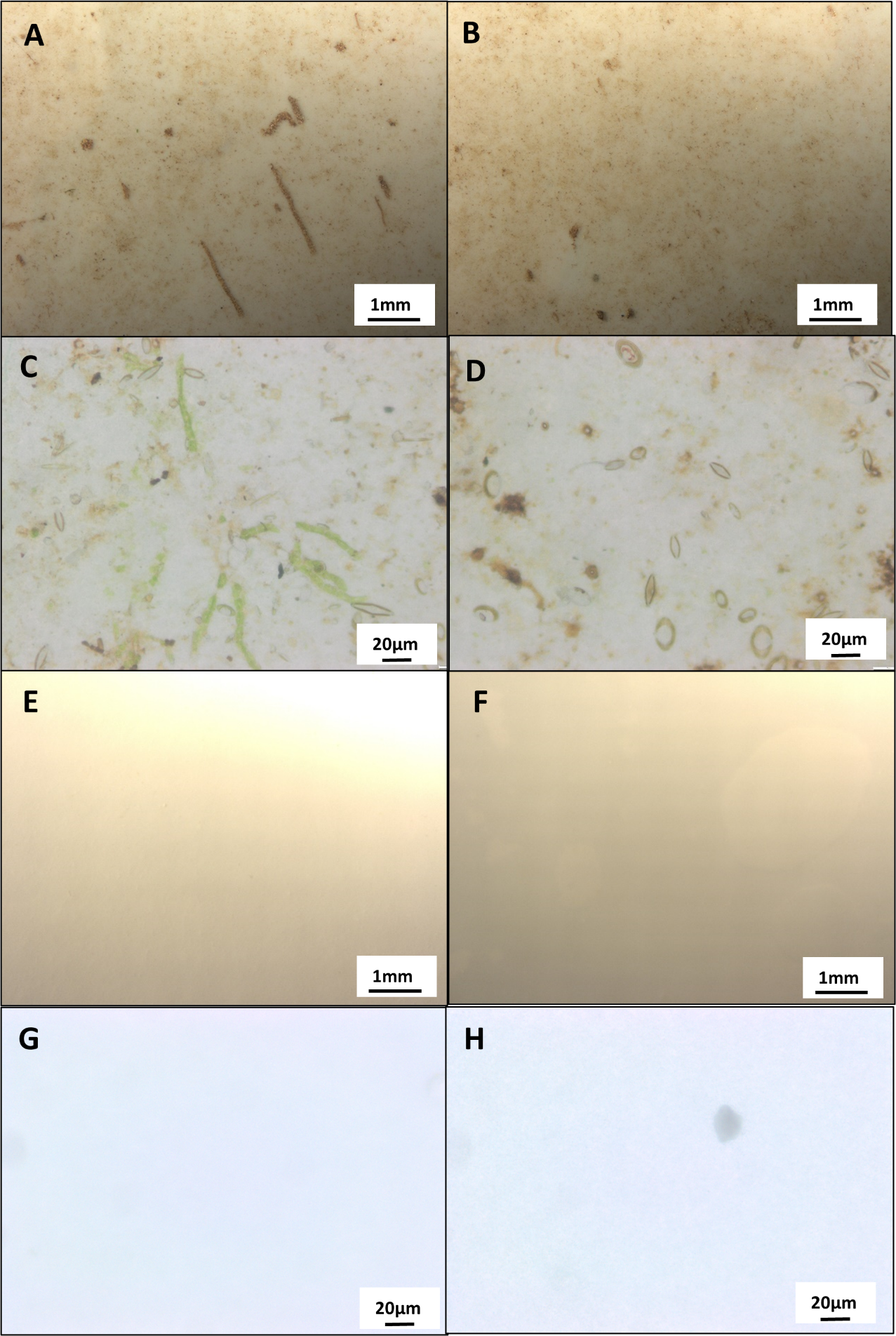
**Figure S4** Images of the surface of LDPE coupons used in Gammarus feeding and behaviour experiments. Colonised plastic which had been incubated in a river for three weeks is shown in images A-D and virgin control plastic soaked in sterile Milli-Q water for three weeks is shown in images E-H.


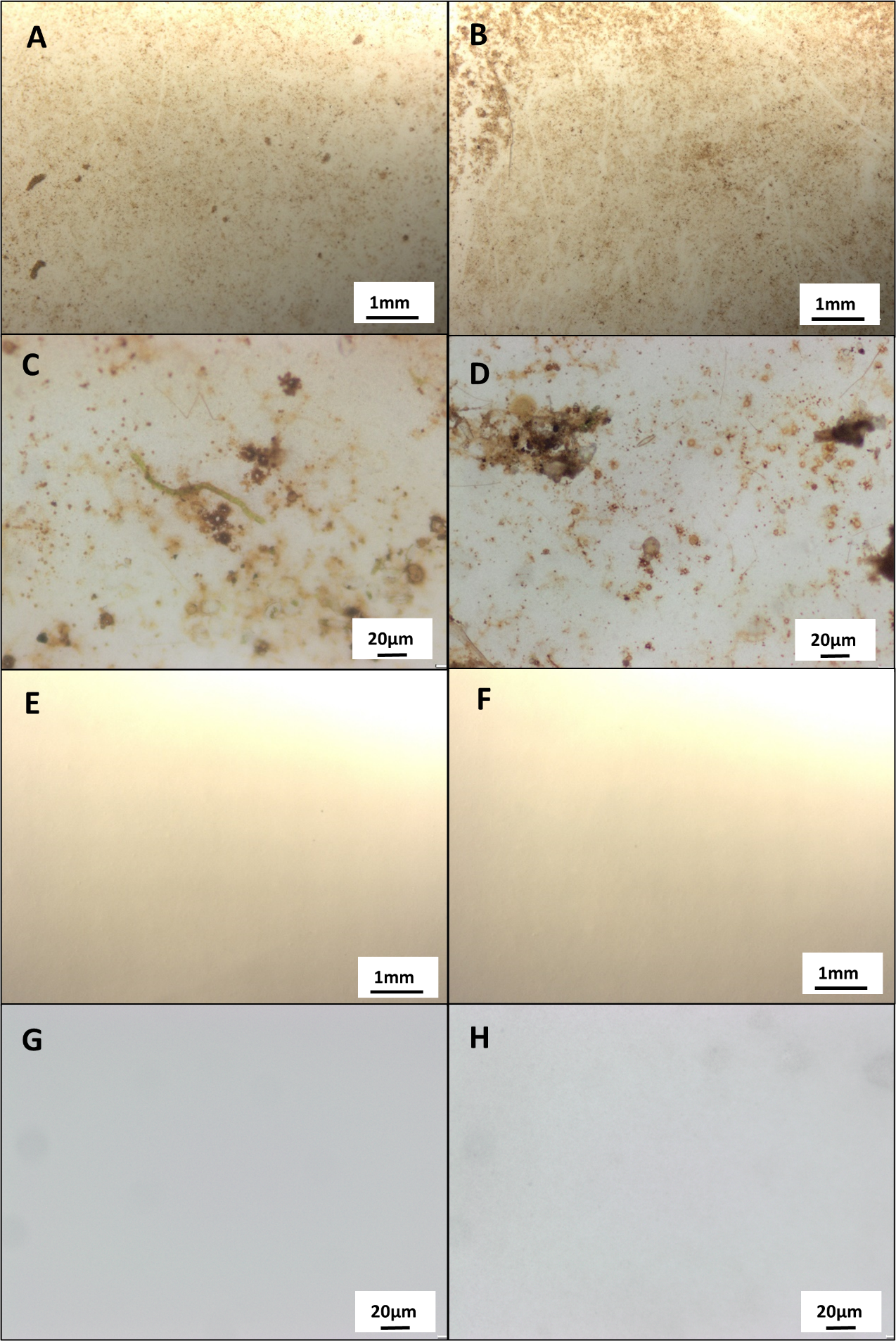
 **Figure S5** Images of the surface of PLA coupons used in Gammarus feeding and behaviour experiments. Colonised plastic which had been incubated in a river for three weeks is shown in images A-D and virgin control plastic soaked in sterile Milli-Q water for three weeks is shown in images E-H

**Table S6** Average disc area ± standard deviation of virgin and colonised LDPE and PLA discs after five days exposure to either control conditions (with no *Gammarus pulex* present) or under treatment conditions with one *Gammarus pulex* individual present.

| Treatment | Average area of disc after exposure ± standard deviation |
| --- | --- |
| Virgin LDPE – Control (No Gammarus) | 751.1 ± 3.2 |
| Virgin LDPE – Exposed to Gammarus | 746.3 ± 8.7 |
| Colonised LDPE – Control (No Gammarus) | 740.2 ± 7.3 |
| Colonised LDPE – Exposed to Gammarus | 748.3 ± 11.9 |
| Virgin PLA – Control (No Gammarus) | 730.7 ± 7.5 |
| Virgin PLA – Exposed to Gammarus | 734.7 ± 5.6 |
| Colonised PLA – Control (No Gammarus) | 744.9 ± 12.6 |
| Colonised PLA – Exposed to Gammarus | 745.2 ± 10.1 |

**Table S7** Water quality parameters of collected river water used throughout the study.

| Water parameter | Value |
| --- | --- |
| Temperature | 15°C |
| pH | 8.04 |
| Alkalinity | 101 ppm |
| Conductivity | 439 (µs/cm) |

Huppertsberg, S., Knepper, T.P., 2020. Validation of an FT-IR Microscopy Method for the Determination of Microplastic Particles in Surface Waters. MethodsX 100874. https://doi.org/10.1016/j.mex.2020.100874

Yuniarto, K., Purwanto, Y.A., Purwanto, S., Welt, B.A., Purwadaria, H.K., Sunarti, T.C., 2016. Infrared and Raman studies on polylactide acid and polyethylene glycol-400 blend. Presented at the THE 3RD INTERNATIONAL CONFERENCE ON ADVANCED MATERIALS SCIENCE AND TECHNOLOGY (ICAMST 2015), Semarang, Indonesia, p. 020101. https://doi.org/10.1063/1.4945555
